# Effect of Drying Phase on the Agglomerates Prepared by Spherical Crystallization

**Published:** 2015

**Authors:** Maryam Maghsoodi, Zahra Yari

**Affiliations:** a *Drug Applied Research Center and School of Pharmacy, Tabriz University of Medical Sciences, Tabriz, Iran. *; b *School of Pharmacy, Kermanshah University of Medical Sciences, Kermanshah, Iran.*

**Keywords:** Drying, Agglomerates, Carbamazepine, Tabletting

## Abstract

In this paper, differences in porosity, compressive strength and tablet- forming ability of carbamazepine crystals agglomerated under similar condition, but subjected to different drying temperatures are reported. The agglomerates were prepared by spherical crystallization method and thereafter dried without agitation at different temperature. An increased drying temperature did not affect the shape and structure texture of dried particles and did not cause them to fracture. Drying of particles at higher temperature suppressed the particle contraction as a consequence of fast evaporation and hence produced particles of larger mean diameter, higher porosity and thus lower compressive strength than those dried at lower temperature. Through a relationship with particle porosity, the drying rate also affected the ability of particles to form tablets.

## Introduction

Agglomeration in liquid systems with a wide range of objectives including separation of colloidal particles from a liquid, spherical granulation, removal and recovery of fine solids from liquid wastes, and selective separation of some components in a mixture of particles has gained increasing attention in recent years due to its relative simplicity and ease of operation (1). Knowledge of the fundamental relationship between the properties of the liquid system and the agglomerates forming ability will allow tailoring of crystallization process in a controlled fashion to produce agglomerates with desired characteristics. Moreover, this may also aid in minimizing unwanted agglomeration in other processes (2,3).

The influence of different variables during the agglomeration in liquid phase on agglomerates properties, such as porosity, has been studied extensively in the literature. It appears that, during agitation in a convective mixer, the granules will densify (4) parallel to their growth by coalescence. The mechanical treatment during agitation, such as agitation speed, temperature of system (5), quantity of binder liquid injected and injection mode (6,7), the primary crystal size (6), appear to affect the densification of the agglomerates during their formation. However, the formation of the agglomerate structure is not completely achieved after agglomeration in liquid phase as it has been demonstrated in some of the previous studies (8).

The drying stage can have a strong influence on the final agglomerate structure, hence drying must not be taken into account only as a secondary process. The drying step could be used as another tool to adjust agglomerate size, density, hardness, *etc*.

In literature, discussions on granule densification during agglomeration in liquid phase are often based on measurements of the porosity of dried granules (9). However, the agglomerates might densify further during the drying phase which thus can affect the final porosity of the dry granules. It seems that the question of the relative importance of the agglomeration step and the subsequent drying step for the final agglomerates porosity has not been addressed in the literature. In the production of agglomerates by spherical crystallization, many researchers used different drying techniques, such as open atmosphere (10-13) and conventional hot air oven at various temperatures (14-18). Moreover, the drying time was variable.

This study represents a contribution in this context. In this study, nearly spherical agglomerates, of carbamazepine were prepared by spherical crystallization. After agglomeration, the particles were dried in various temperatures and importance of the drying phase on the porosity, strength and tablet ability of the carbamazepine agglomerates was investigated. 

## Experimental

*Methods*

The agglomeration procedure used in this study was similar to the one that reported previously (19). A solution of 0.5 g of carbamazepine in 10 mL of ethanol was poured into 84 mL of water at 25 ^o^C under stirring at 400 rpm. The stirring continued for 20 min to obtain agglomerates, which were then filtered and dried. Samples were dried at three different temperatures (35, 45, 55 ^o^C) in the dryer and under ambient conditions (25 ^o^C). The weight loss on drying was determined by removing the particles from the drying condition to an analytical balance at given time intervals. Drying was continued until the weight of the particles was constant with time. Relationships (number of samples=2) between liquid content and drying time were established and a drying rate constant (*k*_D_) was calculated as the gradient of the relationship between ln *m*_t_/*m*_0_ and time*, i.e*.

 Equation (1) $$\ln\frac{m_{t}}{m_{0}}=K_{D}t$$

Where *m*_t_ and *m*_0_ are the amount of removable liquid at time *t *and the total amount of removable liquid, respectively (20). In the other series of drying experiments, the particles were dried without interruption for weighing. These particles were used for porosity determination and tabletting experiments. After drying, all particles were stored in a dessicator over a saturated solution of sodium iodide (40% relative humidity at 20 °C according to Nyqvist (21) before porosity determination and tabletting.

*The agglomerates characterization*

*The particle size and Sphericity*

Light microscopy pictures of the agglomerates (at least 60 particles) are captured by a digital camera and subsequently analyzed by an image analyzing software (scion image analyzer). The average particle size of a single particle is defined as the average length of the distance measured at two degree intervals joining two outline points passing through the center of gravity of the particle. For each particle roundness was determined by the projective image method as follows. Circumference of a projective image and area of a projective image were measured by the scion image analysis software. Roundness was calculated according to Equation (2) (22) 

Equation (2) $${\mathrm{Sphericity}=L}^{2}/4\mathrm{\pi s}$$

Where L = circumference of a projective image and S = area of a projective image. When the *sphericity *of particles was close to 1, the particles closely resembled spherical particles (23). 

*Determination of particle porosity*

The porosity of the particles was calculated from the apparent particle density of carbamazepine powder and the effective particle (particle) density, according to:

Equation (3) $$E\left(\%\right)=(1-\frac{\rho_{e}}{\rho_{a}})\times 100$$

Where ρ_e _and ρ_a _are the effective particle density of the dry particle and the apparent particle density respectively. The apparent particle density (British Standard 2955:1958) of the particles was determined using a helium pycnometer (AccuPyc 1330 Pycnometer, Micromeritics, USA) (number of samples=3). Effective particle density is determined by the projective image count method as follows. The weighed amount of the agglomerates is placed on a glass plate. Heywood diameter and particle number are measured by the scion image analysis software. Subsequently, the effective particle density is calculated according to Equation (4). 

Equation 4 $$\mathrm{effective}\mathrm{particle}\mathrm{density}=\frac{W}{V}=\frac{W}{\sum (\pi d^{3}n/6)}$$

Where W = weight of particles, V = volume of particles, d = Heywood diameter, and n = number of particles (24). 

*Crushing test*

A crushing test in which the agglomerates are crushed between two parallel lateens is used. Thirty agglomerates are randomly removed from the final sample and their size (L) is, measured by optical microscopy and image analysis. Then, each agglomerate is crushed, and the load at failure (m _fail_) is registered. For approximately spherical agglomerate, it is linked to agglomerate size as follows (25) 

Equation (5) $$m_{\mathrm{fail}}=\sigma \frac{\pi L^{2}}{4}$$

Equation (5) is confirmed by a log- log plot of load at failure against agglomerate size. The plot yields the compressive strength (σ). 

*Preparation and characterization of the compacts*

The agglomerates were directly compacted using 8 mm flat-faced punches on a hydraulic press (Riken Seiki Co., Japan). The material for each tablet was weighed (100 mg), introduced into the die and compacted at compression pressures of 200 MPa. The die wall and punch surfaces were lubricated with 1% w/w magnesium stearate in ethanol before compaction. The compacts were held under load for 30 s, ejected and stored in screw-capped bottles for 24 h before using, to allow for possible hardening and elastic recovery. The breaking force (*F*) of the tablets was determined by diametral loading in a standard motorized tablet hardness tester (Erweka, Germany). The tensile strength of the compact was calculated using the following equation (26): 

Equation (6) T=2FπDt

In which D and t are the diameter and thickness of the compact, respectively, and F is the force fracturing the compact. Experiments were repeated five times for statistical reliability and the mean values of five determinations were reported.

*Statistical evaluation of data*

Quantitative data are reported as mean ± standard deviation (SD). Statistical analysis is performed using the analysis of variance (ANOVA). Comparison between the two means is determined using the Tukey’s test with statistical significance evaluated at *P *< 0.05.

## Results

*Effect of drying conditions on drying rate*

Carstensen and Zoglio (20) have suggested that, during tray drying of a bed of granular material, the reduction in liquid content with time should obey a log–lin relationship, *i.e*. ln *m*_t_/*m*_0_ relates linearly to the drying time. 

In this study, log-lin drying profiles of the agglomerates could be described as linear. Thus, the slope values from the relationship between ln* m*_t_/*m*_0_ and the drying time (drying rate constant , K_D_) are used as measures of the drying rate of the agglomerates (Table 1).

Carstensen and Zoglio (20) have suggested that the slope of the log–lin drying rate profile depends on and is inversely related to bed thickness. The thickness of the particle bed was, therefore, kept approximately constant for all the series of granular materials used in this study.

**Table 1:** Drying rate constant, size, circularity, porosity, effective density, compressive strength, of the agglomerates dried at different temperatures and tensile strength of tablets prepared from the agglomerates

**agglomerates dried** **at different temperatures** **(** ^o^ **C)**	**Drying rate constant** ^a^ **(min**^-1^**)**	**Particle** **Size** **d**_ln_**± σ** ** (µm)**	**Sphericity**	**Particle porosity** **(%)**	**Effective** **Particle density** **(g/cm** ^3^ **)**	**Compressive strength ** **(Kg/cm** ^2^ **)**	**Tensile strength of tablets** **(Kg/cm** ^2^ **)**
25	0.11 (0.99)	951± 168	0.89± 0.04	20.3± 0.8	1.03± 0.02	4.75± 0.20	13.8± 1.6
35	0.13 (0.98)	1050± 237	0.88± 0.05	28.6± 0.9	0.92± 0.03	3.99± 0.21	18.9± 1.5
45	0.23 (0.99)	1237± 231	0.87± 0.06	33.8± 1.1	0.86± 0.02	3.28± 0.20	24.3± 1.4
55	0.32 (0.98)	1420± 226	0.88± 0.05	41.6± 0.9	0.75± 0.03	2.72± 0.18	27.1± 1.5

a r^2^- values in parantheses

*Effect of drying rate on shape and porosity of dried particles*

**Figure 1:**
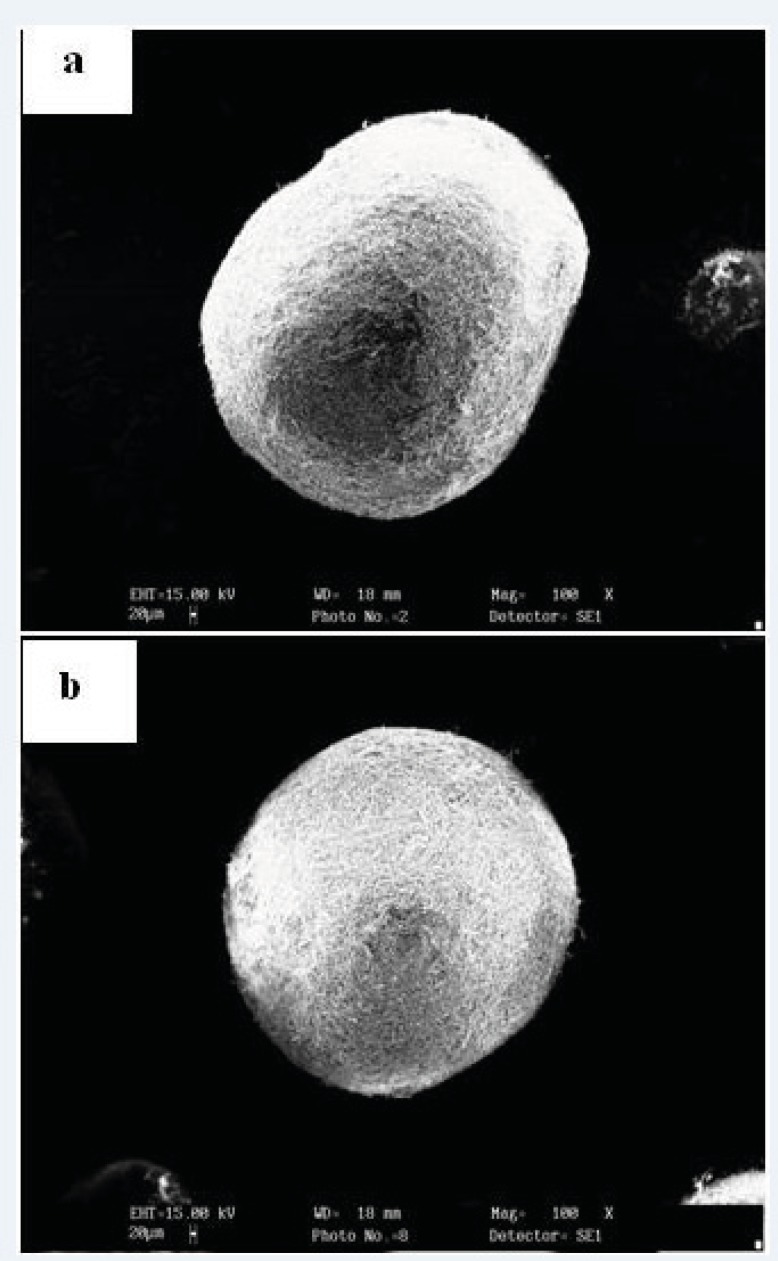
SEM photographs of the agglomerates dried with the highest (a) and the lowest (b) drying rate constant.

It has been reported that fracturing of agglomerates may occur during drying and that the drying stresses developed within the agglomerates, which can explain such fracturing, may vary according to the drying rate (27). The dry particles were, therefore, characterized in terms of shape and appearance. Visual examination of the particles by light microscopy indicated that the particles had generally smooth surface (Figure 1). This inspection did not reveal any cracks on the surface of the particles. In addition, there did not appear to be a general effect of drying rate on the appearance of the particles. The sphericity values (Table 1) confirmed that the shape of the particles was not affected by the drying rate.

According to results, after drying at different rates, the final porosity of the particles was markedly different (Table 1). 

**Figure 2:**
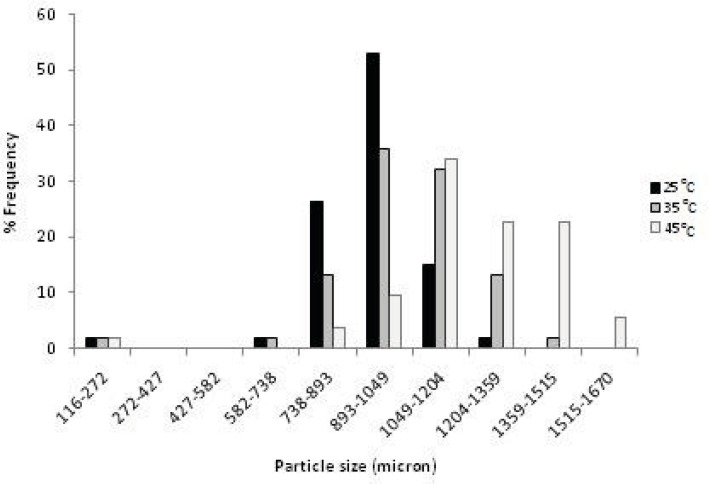
The distribution histograms of the agglomerates dried at different temperatures

The effect of drying rate on the final particle porosity can be easily interpreted in terms of an effect of the drying rate on particle contraction during drying. Comparing the size of the dried agglomerates at different temperature, showed that an increase in the drying rate led to larger agglomerates (Figure 2 and Table 1). In other words, an increased drying rate gave more porous particles, due to decreased particle contraction during the drying process as described below. 

The capillary forces and subsequent compression stresses developed within agglomerate are a result of pressure gradients in the liquid in the pores (27-29). 

It has been proposed that an increased drying rate increases the driving force for contraction *i.e.* the pressure gradient in the liquid is increased (29).

 On the other hand, it can be assumed that the liquid is descending into the pores during the drying phase, *i.e.* at least for a fraction of the pores. The formation of dry pore surfaces may counteract contraction and thus represent a counteracting force towards contraction. The drying behavior will thus depend on the balance between the driving and the counteracting forces for contraction. It can be hypothesized that the formation of dry pore surfaces is dependent on drying rate in such a way that the evaporation of liquid is not balanced

by a contraction of the pore system, *i.e*. drying and subsequent contraction involves a time factor. A possible reason is that all pores of the agglomerate is not emptied in parallel, *i.e*. the larger pores will be emptied first and liquid will flow from the larger pores to the smaller ones that have a higher capillary pressure. It cannot be excluded that with increased drying rate, evaporation will be faster than internal liquid flow which leads to lower contraction during drying. As expected, increasing drying rate led to the agglomerates with lower density as a consequence of lower contraction of particles. 

On the other hand, as indicated in Table 1, there is a clear correlation between particle density and force needed to break the particle. 

Blandin *et al.* (7) have shown that the compressive strength of the agglomerates is due to the two contributions: first, a mechanical contribution due to the very tight piling up of the particles inside the agglomerates and second, the contribution of crystalline bridges. 

Therefore, it is reasonable to assume that piling up the primary crystals more compactly in denser agglomerates results in higher mechanical contribution and consequently higher compressive strength of particles.

On the other hand, solid bridges formation occurred during agglomeration but can be continued upon drying. According to literature, final bridge microstructure is not obtained immediately and develop from a mostly non-crystalline (liquid) and amorphous state to a crystalline structure (30, 31) which is time consumer in the order of hours to days. It is reasonable to assume that development of solid bridge at various drying rate may be different. This fact can thus be account as another reason why the strength of the agglomerates dried at different temperature varies considerably.

It is should be mentioned that reported standard deviations for the compressive strength of the agglomerates are relatively high which may be result of brittle structure of the agglomerates. Because it has been shown that Brittle breakage is difficult to measure quantitatively and reproducible (32).

*Tableting*

As a means of investigating the significance of drying-related contraction behaviour and subsequent differences in porosity of particles, the particles were characterized in terms of tablet-forming ability. Earlier experience of the tabletting behaviour of granules (33) has shown that, for granules compacted at a given applied pressure, granule porosity will be critical for the structure and tensile strength of the formed tablets. These observations were explained as an effect of porosity on the degree of deformation, which the particles undergo during compression. In this study also the porosity of the particles affected the tensile strength of the tablets formed at an applied pressure of 200 MPa (Table 1) in such a way that tablet strength increased with increased particle porosity. In fact a difference in drying rate may significantly affect the tensile strength of tablets formed from agglomerates, modulated by a change in particle porosity. It is possible that a variation in the moisture content of the agglomerates can explain the influence of drying rate on the strength of the tablets (34, 35). In this study, however, the agglomerates were conditioned before tabletting and can thus be assumed to have had similar moisture content, *i.e.* the differences in particle compactability cannot be explained by differences in the moisture content of the particles.

Drying may in addition affect the shape and surface structure of particles. Such an effect may also explain the reported observations, but the characterisation performed in this study indicates that the different drying temperature did not generally affect the shape and surface structure of the particles. Thus, the drying-related change in the tabletting behaviour of the agglomerates is most probably explained by a change in the porosity of the agglomerates, due to differences in their contraction during the drying phase (36). 

## Conclusion

In present study, the effect of drying rate on the contraction of carbamazepine agglomerates and the importance of contraction for the compactability of the agglomerates were studied. Drying rate did not significantly affect the shape of the dried agglomerates and did not cause fracturing of them. An increased drying temperature and consequently drying rate gave more porous agglomerates as a result of decreased particle contraction during the drying. 

Owing to a strong effect of porosity on particle compactability, marked changes in tablet tensile strength with variations in drying rate were obtained. The difference in drying behavior of the dried agglomerates at different rate can be explained by balance between driving force for contraction (pressure gradient in liquid) and the contraction counteracting force (caused by evaporation rate ). 
